# Ezetimibe Induces Paraptosis through Niemann–Pick C1-like 1 Inhibition of Mammalian-Target-of-Rapamycin Signaling in Hepatocellular Carcinoma Cells

**DOI:** 10.3390/genes15010004

**Published:** 2023-12-19

**Authors:** Yuting Yin, Chun Wu, Yufeng Zhou, Meiyin Zhang, Shijuan Mai, Minshan Chen, Hui-Yun Wang

**Affiliations:** 1 State Key Laboratory of Oncology in South China, Sun Yat-Sen University Cancer Center, 651 Dongfeng East Road, Guangzhou 510060, China; yinyt@sysucc.org.cn (Y.Y.); wuchun@sysucc.org.cn (C.W.); zhouyuf@sysucc.org.cn (Y.Z.); zhangmy@sysucc.org.cn (M.Z.); maishj@sysucc.org.cn (S.M.); 2 Guangdong Provincial Clinical Research Center for Cancer, Sun Yat-Sen University Cancer Center, Guangzhou 510060, China; 3 Department of Liver Surgery, Sun Yat-Sen University Cancer Center, Guangzhou 510060, China; chenmsh@sysucc.org.cn

**Keywords:** ezetimibe, paraptosis, ROS, endoplasmic reticulum stress, mTOR

## Abstract

Currently, hepatocellular carcinoma (HCC) is characterized by its unfavorable prognosis and resistance to conventional chemotherapy and radiotherapy. Drug repositioning, an approach aimed at identifying novel therapeutic applications for existing drugs, presents a cost-effective strategy for developing new anticancer agents. We explored the anticancer properties of Ezetimibe, a widely used oral lipid-lowering drug, in the context of HCC. Our findings demonstrate that Ezetimibe effectively suppresses HCC cell proliferation through paraptosis, an apoptotic-independent cell death pathway. The examination of HCC cells lines treated with Ezetimibe using light microscopy and transmission electron microscopy (TEM) showed cytoplasmic vacuolation in the perinuclear region. Notably, the nuclear membrane remained intact in both Ezetimibe-treated and untreated HCC cell lines. Probe staining assays confirmed that the cytoplasmic vacuoles originated from dilated endoplasmic reticulum (ER) compartments rather than mitochondria. Furthermore, a dose-dependent accumulation of reactive oxygen species (ROS) was observed in Ezetimibe-treated HCC cell lines. Co-treatment with the general antioxidant NAC attenuated vacuolation and improved cell viability in Ezetimibe-treated HCC cells. Moreover, Ezetimibe induced paraptosis through proteasome activity inhibition and initiation of the unfolded protein response (UPR) in HCC cell lines. In our in vivo experiment, Ezetimibe significantly impeded the growth of HCC tumors. Furthermore, when combined with Sorafenib, Ezetimibe exhibited a synergistic antitumor effect on HCC cell lines. Mechanistically, Ezetimibe induced paraptosis by targeting NPC1L1 to inhibit the PI3K/AKT/mTOR signaling pathway. In conclusion, our study highlights the potential of Ezetimibe as an anticancer agent by triggering paraptosis in HCC cells.

## 1. Introduction

Paraptosis, a distinct form of programmed cell death from apoptosis, is characterized by the absence of caspase protease family activation and typical apoptotic features such as cell shrinkage, membrane blebbing, and chromatin condensation (pyknosis) [1]. Paraptosis can be triggered by multiple signaling pathways such as mitogen-activated protein kinase (MAPK), mammalian target of rapamycin (mTOR) [2], and phosphatidylinositol 3 kinase (PI3K) [3,4]. The mTOR complex mainly consists of two components, mTOR complex 1 (mTORC1) and mTOR complex 2 (mTORC2). MTORC1 regulates cell growth and metabolism [5], while mTORC2 promotes cell growth [6]. Apart from this, another mTOR complex regulates cell proliferation and migration through ribosomal protein S6 kinase (S6K) and eIF4E-binding protein (4E-BP1) [7,8]. The central mechanism underlying paraptosis involves the generation of reactive oxygen species (ROS). The cell viability is decreased in cancer cells and extensive cytoplasmic vacuolation occurs [9]. Paraptosis has been seen in cells infected with the Zika virus [4], dying retinal pigment epithelial cells caused by corticosteroids [10], and retinal ganglion cells in early-stage glaucoma [11]. In addition, paraptosis has been proposed as a potential mechanism for eliminating cells with defective apoptotic programs [12]. Morphologically, paraptosis is characterized by swelling of the endoplasmic reticulum (ER) and mitochondria, along with cytoplasmic vacuolization, while maintaining membrane integrity. As paraptosis progresses, membrane integrity is lost, resulting in “implosive” destruction and the formation of bubble-like membrane expansions [4]. ER architectural disruption leads to dilatation and widespread vacuolation [13], with proteasomal dysfunction primarily responsible for ER dilation and vacuolation [14]. Imbalances in calcium ions (Ca^2+^) between the mitochondria and ER, as well as the unfolded protein response (UPR), are also implicated in vacuole formation [15]. On another note, several natural compounds have demonstrated the ability to induce paraptosis either independently or in synergy with apoptosis-inducing drugs, suggesting that strategies targeting paraptosis may overcome primary drug resistance in cancer cells. Hence, compounds capable of inducing paraptosis hold promise as anti-cancer drugs.

Ezetimibe, an FDA-approved drug, is utilized to reduce blood cholesterol levels. Its mechanism of action involves the inhibition of the Niemann–Pick C1-Like 1 (NPC1L1) protein, which diminishes cholesterol absorption in the intestine, specifically at the luminal membrane of intestinal epithelial cells [16]. Consequently, this reduction lowers the risk of cardiovascular events [17]. NPC1L1 also modulates the absorption of various lipid-soluble nutrients in the intestine. In a recent study by Zhang et al., RNA-seq analysis was employed to identify gene expression differences between multidrug-resistant (MDR) persister cancer cells and MDR cancer cells. This study discovered that NPC1L1 is highly expressed in MDR persister cancer cells and functions as a crucial effector of oxidative stress [18]. It was proposed that by generating oxidative stress-dependent lethal macropinocytosis, a combination of routine anti-MDR drugs with Ezetimibe effectively lowers the pool of remaining persister cells in MDR cancer cells, thus positioning Ezetimibe as a potential antitumor drug.

In our current study, we have uncovered that Ezetimibe has the ability to induce paraptosis in hepatocellular carcinoma (HCC) cells through mechanisms involving endoplasmic reticulum (ER) stress, the unfolded protein response (UPR), and activation of the PI3K/AKT/mTOR and MAPK/JNK signaling pathways. Moreover, our findings demonstrate that the simultaneous treatment of Ezetimibe and Sorafenib synergistically inhibits the growth of HCC cells.

## 2. Materials and Methods

### 2.1. Cell Lines and Cell Culture

HepG2, SNU-449, and Huh7 cells were obtained and maintained in our laboratory. The authenticity of these cell lines was confirmed by the ATCC Cell STR Testing Service (last tested on 20 May 2021, Beijing Zhongyuan Ltd., Beijing, China) to ensure their validity. Upon revival, the cell lines were cultured for a maximum of 5 passages in DMEM (Invitrogen, Waltham, MA, USA) or RPMI 1640 Medium (Invitrogen) supplemented with 10% fetal bovine serum (FBS) (Biological Industries, Beit Haemek, Israel). A 5% carbon dioxide humidified chamber was used for incubating all cells at 37 °C.

### 2.2. Plasmid Transfection

A 6-well plate was used to cultivate the cells until they were 70% to 80% confluent. Plasmid transfections were performed using Lipofectamine 3000 (Invitrogen). Regular testing was conducted using the LookOut Mycoplasma PCR Detection Kit (MP0035; Sigma, St. Louis, MO, USA) to ensure the absence of mycoplasma contamination. Subsequently, a transfection mixture was prepared by combining 6 μL of Lipofectamine 3000 (Invitrogen, Carlsbad, CA, USA), 125 μL of Opti-MEM medium, and 5 μL of the desired expression plasmid at room temperature. Then, 125 μL of this transfection mixture was added to the cultured cells in the 6-well plate and incubated for 48 h. The efficiency of overexpression was confirmed by performing Western blotting analysis.

### 2.3. Measurement of Cell Viability

For seeding the cells, 96-well plates were utilized, allowing them to grow for 24 h. Subsequently, the cells were treated with either vehicle or various doses of Ezetimibe for different durations. Cell viability was evaluated using a Cell Counting Kit-8 (CCK-8).

### 2.4. Colony Formation Assay

In this experiment, 150 cells were seeded in each well of a 6-well plate and cultured for a period of 2 weeks. Once visible clones had formed, the clones were fixed by adding 75% alcohol to each well and incubating for 20 min. After fixation, the clones were stained with 0.5% crystal violet solution for an additional 20 min. By multiplying the number of clones by the number of originally seeded cells, we calculated the clone formation rate. This experiment was repeated twice to ensure the reliability and consistency of results.

### 2.5. Microscopy, Electron Microscopy, and Confocal Laser Scanning Microscopy

A 4% paraformaldehyde solution was used to fix cells for immunofluorescence studies after they were cultured on coverslips for the indicated periods. Olympus, Tokyo, Japan, provided an inverted microscope that captured fluorescence and differential interference contrast images (DIC). Laser scanning microscopes (Zeiss, Jena, Germany) were used to obtain confocal images. Photographs were taken using an inverted microscope (Nikon, Tokyo, Japan).

In the electron microscopy analysis, cells were fixed in 2% glutaraldehyde and washed in 0.1 M phosphate-buffered saline. Osmium tetroxide was used as a secondary fixative. Following dehydration in different concentrations of acetone, the cells were embedded in epoxy resin (EM Sciences, Hatfield, PA, USA). We mounted ultrathin sections of 50–60 nm on copper grids and stained them with lead citrate and uranyl acetate.

After treatment with different concentrations of Ezetimibe, SNU-449 and HepG2 cells were incubated with ER-tracker Red (BODIPY TR, C1041S-1) and Mito Tracker Green (Molecular Probes, C1048) at 37 °C for 15 min in the dark. After washing the cells with phosphate buffer solution (PBS), each well was treated with 50 μL of DAPI staining solution. The morphology of organelles was observed using confocal laser scanning microscopy.

### 2.6. EdU Incorporation Assay

The EdU incorporation assay on HCC cells was conducted using the BeyoClick™ EdU Cell Proliferation Kit with Alexa Fluor 488 (Beyotime, Shanghai, China). After treatment with Ezetimibe at concentrations ranging from 0 to 75 μM for 24 h, cells were labeled with 5-ethynyl-2′-deoxyuridine (EdU) and stained with Hoechst 33342. Images were captured using an Olympus FSX100 microscope (Olympus, Tokyo, Japan).

### 2.7. 20S Proteasome Activity Assay

A 24 h treatment with Ezetimibe was followed by harvesting, washing, and lysing the cells in RIPA lysis buffer (Beyotime) without protease inhibitors. Using a BCA protein assay kit (Beyotime), equal portions of protein, fluorescent substrate (Suc-Leu-Leu-Val-Tyr-AMC), and buffer were added to the wells of a black 96-well plate with a transparent flat bottom. Subsequently, a proteasome inhibitor, MG-132 (1 μM) or vehicle, was added. Using a fluorescence microplate reader, the liberated AMC was measured after 1 h of co-incubation at 37 °C.

### 2.8. Apoptosis Analysis

SNU-449 and HepG2 cells were plated in 6-well plates. After 24 h, various concentrations of Ezetimibe were added to the wells. After a 48 h treatment period, apoptosis kit (BD Biosciences, San Jose, CA, USA) was used to stain the cells with 5 mg of Annexin V-APC and 7 mg of 7-AAD. With an FACS CaliburTM (BD Biosciences) flow cytometer, we examined the cells’ apoptosis.

### 2.9. Reverse Transcription and Quantitative Real-Time PCR (qRT-PCR)

TRIZOL (Invitrogen) was used to extract total RNA from cells. Subsequently, the GoScriptTM Reverse Transcription System kit (Promega, Madison, WI, USA) was used to synthesize cDNA from total RNA samples. The GoTaq RT-PCR Master Mix (Promega) was used for quantitative real-time PCR. A primary denaturation stage at 95 °C for 10 min was followed by 40 cycles of amplification, which included denaturation for 15 s at 95 °C and annealing at 60 °C for 1 min. The final step involved denaturation at 95 °C for 30 s. This process was carried out on a Roche Lightcycler 96 RT-PCR system (Roche Diagnostics). The primer sequences of NPC1L1: forward, 5′-AGAGCTAGCGAATTCGCCACCATGGCGGAGGCCGGCCTGA-3′; reverse, 5′-CAGCGGCCGCGGATCCAGCGTAGTCTGGGACGTCGTATGGGTAGAACTGCCGCCCATTGTTGG-3′. The primer sequences of GAPDH: forward, 5′-GAAGGTGAAGGTCGGAGTC-3′; reverse, 5′-GAAGATGGTGATGGGATTTC-3′. To perform relative quantification, ΔΔCt values [19] were determined by utilizing GAPDH as a control standard within each sample. To create a visual representation, these values were adjusted to match the controls.

### 2.10. Western Blot Analysis

Lysis buffer was prepared using RIPA lysis buffer (CWBio Co., Ltd., Cambridge, MA, USA) containing 1% protease and phosphatase inhibitors (Thermo Scientific, Waltham, MA, USA). The protein concentration in the lysates was determined using a BCA Protein Assay Kit (Thermo Scientific). For protein analysis, electrophoresis of 20 g of the protein samples on a 10% SDS-PAGE gel was followed by transfer to PVDF membranes (0.45 m; Merck Millipore; Bio-Rad, Hercules, CA, USA) using a wet transfer system. Incubation with primary antibodies against specific proteins was carried out overnight at 4 °C after blocking with 5% skimmed milk for one hour at room temperature. The primary antibodies used included those against GAPDH (CST, #5174), p-mTOR (CST, #5536), mTOR (CST, #2972), p-AKT (CST, #4060), AKT (CST, #4691), p-PI3K (CST, #17366), PI3K (CST, #4292), p-S6K1 (CST, #9234), S6K1 (CST, #34475), p-JNK (CST, #4668), JNK (CST, #9252), p-MAPK (CST, #4511), MAPK (CST, #8690), p-MEK (CST, #2338), MEK (CST, #91222), p-PERK (CST, #3179), ERK (CST,#5683), CHOP (CST,#2895), BIP (CST,#63411). Subsequently, the membranes were incubated with secondary antibodies for an additional hour. Finally, the protein bands on the blots were visualized using an enhanced chemiluminescence (ECL) system (Millipore, Burlington, MA, USA).

### 2.11. In Vivo Tumor Xenograft Studies

The mouse experiments conducted and in vivo tumor xenograft studies followed the approved protocols by the Animal Care and Use Committee of Sun Yat-Sen University Cancer Center (SYSUCC), with specific protocol ID: L102012022040C. BALB/c nude mice aged 4–6 weeks old were obtained from the Foshan Medical Laboratory Animal Center in Guangdong Province, China. Huh-7 cells were subcutaneously injected into the left flank of each mouse at a concentration of 1 × 106 cells. Once the average tumor volume reached approximately 50 to 100 mm^3^, the mice were randomly assigned to one of four treatment groups: control (vehicle), Ezetimibe 10 (10 mg/kg dosage), Ezetimibe 20 (20 mg/kg), and Ezetimibe 30 (30 mg/kg). An oral administration of Ezetimibe in DMSO and saline solution was conducted for 17 days. Tumor volume was measured daily using calipers and calculated using the formula V = length × width^2^/2. For further analysis, tumor tissues were extracted from the mice, weighed, and resected after euthanasia.

### 2.12. Statistical Analyses

Statistical analysis of the data in this study was conducted using GraphPad Prism 7. The values presented are the mean + standard deviation (SD) of three independent experiments. Student’s *t*-test was used to compare means between two groups, and one-way Analysis of Variance (ANOVA) was performed for comparisons among multiple groups. *p* values less than 0.05 were considered significant.

## 3. Results

### 3.1. Ezetimibe Treatment Induces Cell Death That Is Independent of Apoptosis

**Figure 1:**
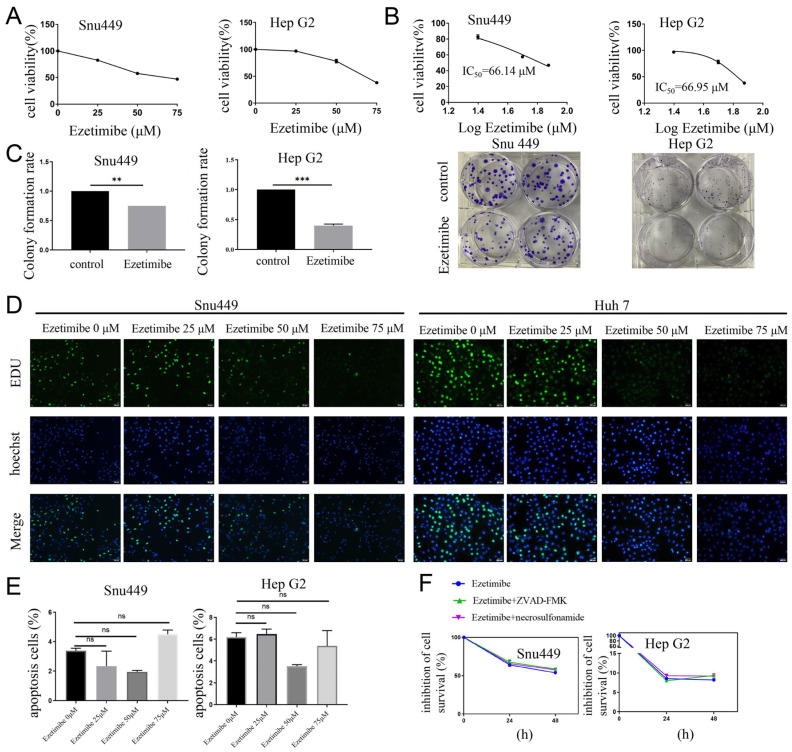
Ezetimibe inhibits proliferation of HCC cells. (**A**) Cell viability is gradually decreased in HCC cells treated with different concentrations of Ezetimibe for 48 h. Using CCK8 assay, cell viability was determined. (**B**) IC50s of Ezetimibe in SNU-449 and HepG2 cells were calculated. (**C**) Colony formation assay was performed on SNU-449 and HepG2 cells treated with Ezetimibe (75 μM) or vehicle (control). Cells (3 × 10^2^) were seeded in the 6-well plate with or without Ezetimibe. After the 3rd day, culture medium was exchanged for new medium and colonies were counted on the 14th day after the cells were fixed and stained with 0.1% crystal violet. ** *p* < 0.01; *** *p* < 0.001. (**D**) EDU staining was used to measure the cellular proliferation based on the reaction with Alexa488-azide (green) at 48 h. Scale bars, 50 μM (SNU-449 cells) and 200 μM (Huh7 cells). (**E**) Apoptosis was analyzed using flow cytometry on HCC cells treated with Ezetimibe ranging from 0 to 75 μM for 24 h in 6-well plates. (**F**) Cells were treated with Ezetimibe (75 μM)) as well as ZVAD-FMK (20 μM) and necrosulfonamide (0.5 μM). The cell viability was measured according to CCK8.

In this study, the potential inhibitory effect of Ezetimibe on the proliferation of HCC cell lines was investigated for drug repositioning. HCC cell lines (SNU-449, HepG2, Huh7, and Hep-3B cell lines) were treated with various doses of Ezetimibe ranging from 0 to 75 μM. After a 48 h treatment period, cell viability was assessed using the CCK-8 assay. The results demonstrated that the cell viability of these HCC cells was inhibited by more than 50% at a concentration of 75 μM (Figure 1A and Figure S1A). The calculated 50% inhibitory concentration (IC50) values for Ezetimibe were found to be 66.14 μM for SNU-449 cells and 66.95 μM for HepG2 cells (Figure 1B). In the colony formation experiment conducted over a 2-week period, IC50 doses of Ezetimibe resulted in a decrease of 30% and 50% in SNU-449 and HepG2 cells, respectively. In addition, the number of colonies formed from Ezetimibe-treated HCC cells was significantly lower than that from control HCC cells (Figure 1C). Furthermore, an EdU Cell Proliferation assay was performed to examine the incorporation of EdU into genomic DNA during cell proliferation after a 48 h treatment period. The results demonstrated that HCC cells treated with higher doses (50 or 75 μM) of Ezetimibe displayed fewer or weaker EdU-positive cells compared to those treated with lower doses (0 or 25 μM) of Ezetimibe (Figure 1D and Figure S1B,C), suggesting the decrease in proliferation in HCC cell lines. To determine whether the inhibition of HCC cells by Ezetimibe is mediated through apoptosis, Annexin V-APC/7-AAD staining and flow cytometry were performed on SNU-449 and HepG2 cells treated with various doses (0 to 75 μM) of Ezetimibe for 48 h. The results indicated that there were no significant differences in the rates of apoptosis among these HCC cells treated with or without different doses of Ezetimibe (Figure 1E), suggesting that apoptosis is not the mechanism responsible for the suppression of cell proliferation and growth of HCC cell lines by Ezetimibe. Interestingly, ZVAD-FMK (an apoptosis inhibitor) and necrosulfonamide (a necroptosis inhibitor) had no effect on Ezetimibe-induced cell death (Figure 1F). These findings suggest that Ezetimibe can induce HCC cell death through a mechanism other than apoptosis.

### 3.2. Ezetimibe Produces Massive Cytoplasmic Vacuolation in HCC Cells

**Figure 2:**
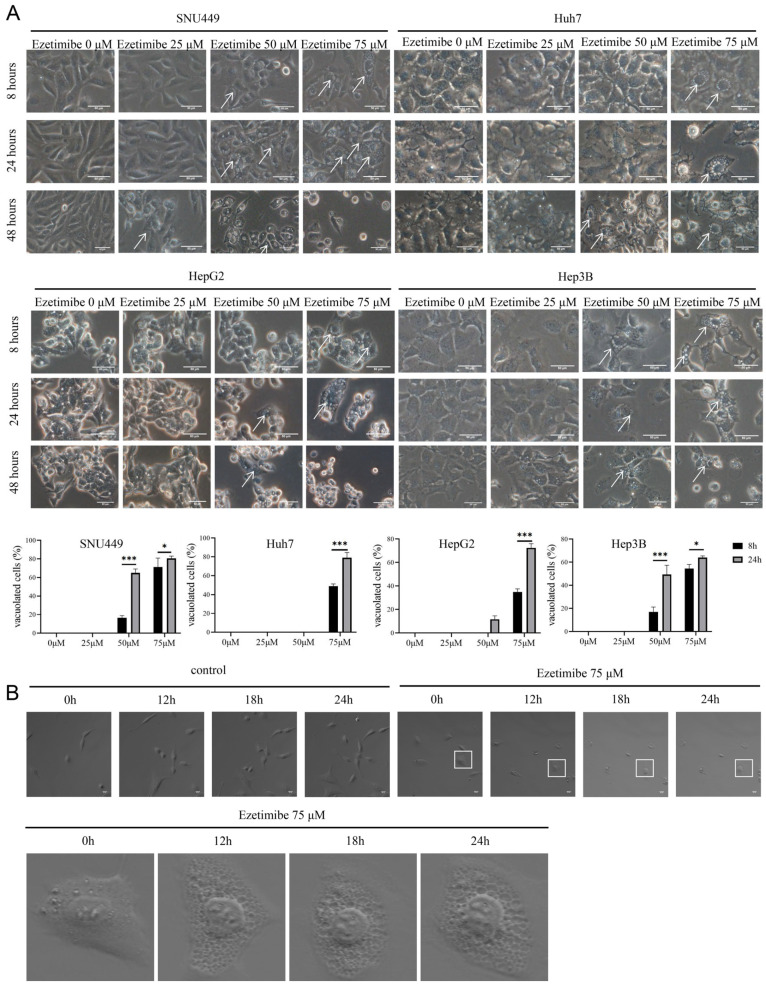
Ezetimibe treatment induces massive cytoplasmic vacuolation. (**A**) HCC cells were treated with Ezetimibe ranging from 0 to 75 μM for 8–24 h. Perinuclear vacuoles were observed in HCC cells with 50 μM Ezetimibe at the 8th hour and massive vacuoles in HCC cells with 75 μM Ezetimibe at the 24th hour using a 20× objective. Quantification of vacuolated cells. * *p* < 0.05, *** *p* < 0.001. (**B**) SNU-449 cells treated with or without Ezetimibe (75 μM) for 24 h were examined by live cell imaging. Scale bars, 50 μm. The cells were magnified.

The involvement of cell death processes in the anticancer effect of Ezetimibe was investigated in various HCC cell lines, including SNU-449 cells, Huh 7 cells, Hep-3B cells, and HepG2 cells. Cells were treated with Ezetimibe at varying concentrations (0 to 75 μM) for 8 or 24 h. Cytoplasmic vacuolation and subsequent cell death were observed as Ezetimibe concentrations increased from 0 to 75 μM in SNU-449 and HepG2 cells (Figure 2A, upper panel). A similar cytoplasmic vacuolation and cell death were also observed in Huh7 and Hep-3B cells treated with Ezetimibe at a concentration of 75 μM (Figure 2A, lower panel). The vacuoles appeared small after an 8 h treatment with Ezetimibe but became larger after a 24 h treatment, primarily located in the perinuclear region and cytoplasm. Importantly, intact nuclei were observed in the vacuolated HCC cell lines (Figure 2A), indicating a different process from apoptosis.

The HCC cell lines were examined under a microscope to evaluate the mode of cell death induced by Ezetimibe (Figure 2B). SNU-449 cells that were not treated with Ezetimibe did not exhibit cytoplasmic vacuoles. However, when those same cells were treated with Ezetimibe at a concentration of 75 μM, vacuolation was evident (Figure 2B). These findings suggest that Ezetimibe can induce vacuolation and subsequent cell death in HCC cells.

### 3.3. Ezetimibe-Induced Cell Vacuolization Originates from ER

**Figure 3:**
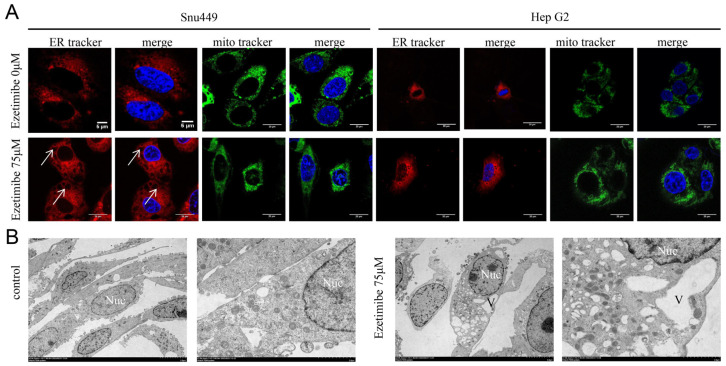
Cytoplasmic vacuoles are derived from endoplasmic reticulum in Ezetimibe-treated cells. (**A**) SNU-449 and HepG2 cells were treated with control or Ezetimibe (75 μM) for 24 h. After being harvested, these cells were stained with ER Tracker (red) and Mito Tracker (Green) and counterstained with DAPI (blue), and then cells were observed using confocal microscopy using a 63× objective. Ezetimibe-induced cytoplasmic vacuoles were co-localized using ER Tracker. Scale bars, 5 μm (SNU-449 cells stained with ER Tracker) and 20 μm (SNU-449 cells stained with Mito Tracker and Huh7 cells). (**B**) SNU-449 cells were treated with Ezetimibe (75 μM) for 24 h, and the cytoplasmic vacuoles were observed under transmission electron microscopy. Scale bars, 2 μm.

We aimed to determine the organelle from which the vacuolation induced by Ezetimibe originates. Based on previous research by [20], it has been established that ER dilatation is a prominent feature of paraptosis-associated cell death. Therefore, we stained HCC cells with ER and mitochondria probes to determine whether vacuoles originated from mitochondria or ER [21,22,23]. As shown in Figure 3A, HCC cells treated with Ezetimibe exhibited vacuoles surrounded only by ER-tracker fluorescence, with no associated Mito-tracker fluorescence. In contrast, untreated HCC cells did not display any vacuoles. These findings strongly suggest that the endoplasmic reticulum is the source of the vacuolation induced by Ezetimibe. To further investigate the origin of the vacuoles, we utilized transmission electron microscopy (TEM). TEM analysis revealed numerous vacuoles with well-defined borders adjacent to the nucleus in Ezetimibe-treated SNU-449 cells (Figure 3B). Importantly, the nuclear membranes remained intact, and no morphological characteristics of typical apoptosis were observed. Additionally, only a few autophagic vacuoles were detected. Furthermore, these findings suggest that Ezetimibe causes cell death through parapoptosis, with vacuoles originating from the endoplasmic reticulum.

### 3.4. Ezetimibe-Induced ER Stress and ROS Generation Result in Paraptosis in HCC Cells

**Figure 4:**
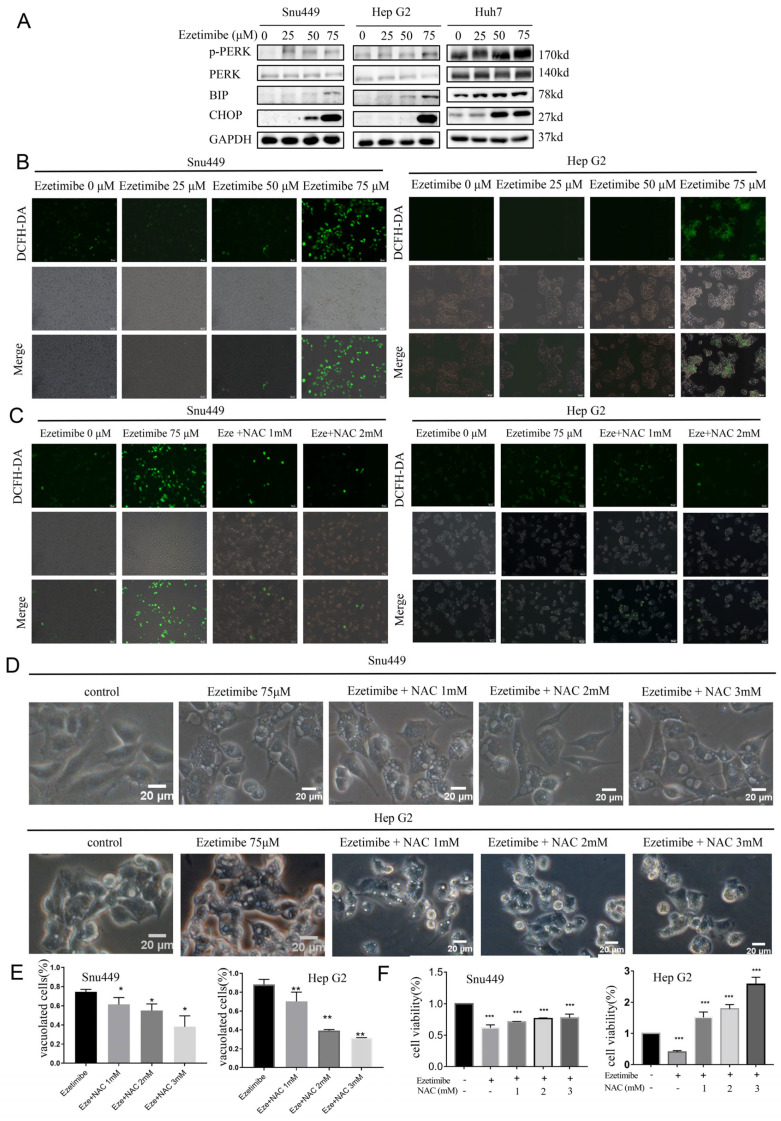
Ezetimibe induces ROS and ER stress in HCC cells. (**A**) The expression levels of proteins related to UPR and ER stress were increased in HCC cells treated with Ezetimibe ranging from 0 to 75 μM for 24 h, as shown by Western blot. (**B**) SNU-449 and HepG2 cells were treated with Ezetimibe ranging from 0 to 75 μM for 24 h and then the cells were stained with DCFH-DA of Cellular ROS Assay Kit (ROS exhibited green fluorescence). (**C**) When SNU-449 and HepG2 cells were treated with Eze (Ezetimibe, 75 μM) and antioxidant NAC from 1 to 2 μM, ROS (green fluorescence) nearly disappeared. (**D**,**E**) SNU-449 cells were treated with Ezetimibe (75 μM) and NAC (1–3 mM), and the perinuclear vacuoles were significantly reduced in the cells treated with 2 or 3 mM NAC for 24 h compared with cells treated with Ezetimibe alone. * *p* < 0.05, ** *p* < 0.01. (**F**) Cell viability inhibited by Ezetimibe (75 μM) was restored by NAC (1–3 mM). Scale bars, 50 μM (**A**,**B**) and 20 μM (**C**). *** *p* < 0.001.

ER vacuolation can be a consequence of prolonged or severe ER stress. Therefore, it is speculated that Ezetimibe induces ER vacuolation by initiating ER stress in HCC cells. Previous reports have demonstrated that Immunoglobulin Heavy Chain-Binding Protein (BIP), an essential chaperone protein located in the ER, assists in protein folding and prevents protein aggregation. During ER stress, BIP is upregulated to alleviate the stress. C/EBP homologous protein (CHOP) is induced during ER stress, and it plays a key role in regulating cell survival and death, including paraptosis. Another key sensor of ER stress is PERK (PKR-like endoplasmic reticulum kinase). Hence, BIP, CHOP, and PERK are considered markers for ER stress [24]. To investigate the speculation that ER stress induces ER vacuolation, the expression of BIP, CHOP, and PERK was assessed using Western blotting after treatment with Ezetimibe at concentrations ranging from 0 to 75 μM. As expected, Ezetimibe treatment significantly increased the levels of BIP, CHOP, and phosphorylated PERK (Figure 4A), indicating that Ezetimibe indeed triggers ER stress and ultimately leads to ER vacuolation.

The accumulation of reactive oxygen species (ROS) has been reported to induce ER stress and lead to paraptosis [25,26]. To investigate whether Ezetimibe can induce ROS accumulation in HCC cells, ROS levels were assessed in HCC cell lines treated with Ezetimibe using a previously reported method [27,28]. SNU-449, HepG2, and Huh7 cells were treated with Ezetimibe (0 to 75 μM). As depicted in Figure 4B and Figure S2, Ezetimibe treatment resulted in a dose-dependent increase in ROS levels (green fluorescence). The accumulation of ROS was diminished by treatment with the general antioxidant N-acetylcysteine (NAC) at concentrations of 1 or 2 mM in SNU-449 and HepG2 cells (Figure 4C). Importantly, the Ezetimibe-induced cytoplasmic vacuolation and cell death could be dose-dependently prevented by treatment with NAC in SNU-449 and HepG2 cells (Figure 4D,E). Furthermore, the cell viability of HepG2 cells treated with NAC at a concentration of 3 mM was higher than that of the control cells [29,30], and the inhibition of cell viability by Ezetimibe was gradually attenuated by NAC in a dose-dependent manner [31] (Figure 4F). Ezetimibe-induced paraptosis is strongly associated with ROS production. In conclusion, the treatment with Ezetimibe induces the generation of ROS, which in turn triggers ER stress and vacuolation, ultimately leading to paraptosis in HCC cells. This process can be prevented by the use of the general antioxidant NAC.

### 3.5. Ezetimibe Induces Paraptosis by Inhibiting Proteasome Activity

**Figure 5:**
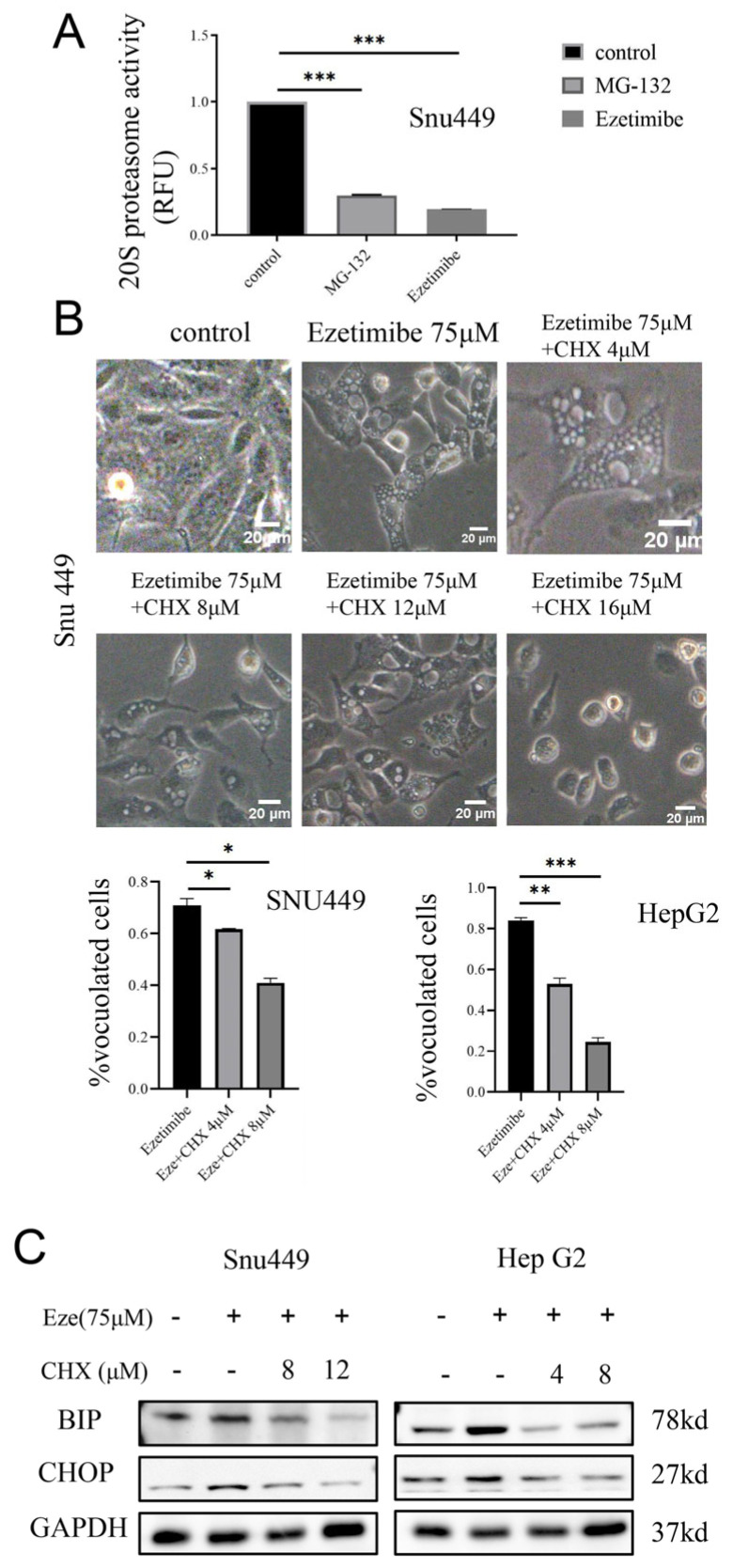
Ezetimibe induced paraptosis by inhibiting activity of proteasome. (**A**) SNU-449 cells were treated with Ezetimibe (75 μM) for 24 h and used to determinate 20S protease activity; the proteasome activity was inhibited by Ezetimibe. MG-132 (1 μM) was used as a positive control. *** *p* < 0.001. (**B**) Cycloheximide (CHX) significantly decreased perinuclear vacuolation induced by Ezetimibe in a dose-dependent manner (4–12 μM) after treatment for 24 h compared with cells treated with Ezetimibe alone. Images were taken with a 20× objective. Scale bars, 20 μM; * *p* < 0.05, ** *p* < 0.01, *** *p* < 0.001. (**C**) CHX decreased the expressions of BIP (ER stress protein) and CHOP, which was detected by Western blot.

Multiple studies have indicated that inhibiting the proteasome can result in ER expansion, activation of the unfolded protein response (UPR), and accumulation of proteins within the ER, ultimately leading to paraptosis [2,23,32,33]. Therefore, we aimed to investigate the impact of Ezetimibe on proteasome activity in HCC cell lines. As expected, Ezetimibe significantly inhibited proteasome activity in SNU-449 cells (Figure 5A), leading to the accumulation of proteins within the cells [34,35,36]. Additionally, the influence of cycloheximide (CHX), a protein synthesis inhibitor, on paraptosis was evaluated. The results demonstrated that the treatment with CHX for 24 h reversed the Ezetimibe-induced vacuolation and cell death in both SNU-449 and HepG2 cells, with concentrations ranging from 4 to 12 μM (Figure 5B and Figure S3). It is important to note that higher doses of CHX resulted in increased cell death due to the toxicity of CHX (16 μM) (Figure 5B). Western blot analysis further confirmed that CHX treatment significantly reduced BIP, a marker of ER stress, and CHOP expression (Figure 5C). Taken together, these findings indicate that Ezetimibe induces ER stress and the accumulation of unfolded and/or misfolded proteins by inhibiting proteasome activity, causing ER enlargement. Furthermore, CHX inhibits protein synthesis in HCC cells, preventing paraptosis.

### 3.6. Ezetimibe Induces Paraptosis by Targeting NPC1L1 to Enhance MAPK Pathway and Inhibit mTOR Pathway in HCC Cells

**Figure 6:**
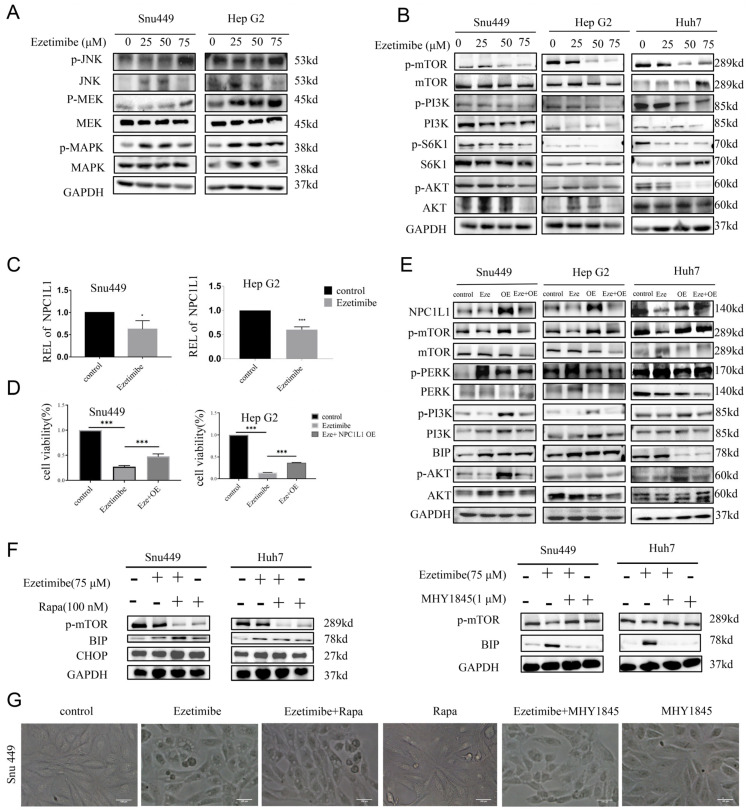
Ezetimibe induces paraptosis via inhibition of mTOR signaling by targeting NPC1L1. (**A**) The expression levels of JNK, MAPK, and MEK proteins related to signaling pathways regulating paraptosis were also enhanced in HCC cells treated with Ezetimibe (0–75 μM) as presented by Western blotting assay. (**B**) The expression levels of mTOR, PI3K, S6K1, and AKT proteins related to signaling pathways regulating paraptosis were decreased in HCC cells treated with Ezetimibe (0–75 μM) as presented by Western blot. (**C**) REL (relative expression level) of NPC1L1 was detected using RT-PCR in HCC cells treated with Ezetimibe or control. * *p* < 0.05; *** *p* < 0.001. (**D**) The CCK8 assay was used to determine the cell viability of HCC cells after treatment with Ezetimibe 75 μM or the over-expression of NPC1L1. *** *p* < 0.001. (**E**) Overexpression of NPC1L1 impacted the expression of proteins involved in ER stress and PI3K/AKT/mTOR signaling. (**F**) The cells were divided into four groups: control, Ezetimibe (75 μM), Ezetimibe + rapamycin (100 nM)/MHY1845 (1 μM), and rapamycin (100 nM)/MHY1845 (1 μM). Expression of p-mTOR, BIP, and CHOP proteins was detected by Western blotting. (**G**) SNU-449 cells were treated with control, Ezetimibe (75 μM), Ezetimibe + rapamycin (100 nM)/MHY1845 (1 μM), and rapamycin (100 nM)/MHY1845 (1 μM) for 24 h. Scale bars, 100 μm.

In general, the activation of the mitogen-activated protein kinase (MAPK) signaling pathway has been shown to participate in paraptosis by promoting ER stress [37,38,39,40]. In HCC cell lines treated with Ezetimibe, expression levels of pathway genes were evaluated to investigate the involvement of MAPK signaling in paraptosis. As anticipated, Ezetimibe treatment significantly increased the expression levels of p-JNK, p-MEK, and p-MAPK (Figure 6A and Figure S4), indicating that the MAPK signaling pathway activated by Ezetimibe upregulates ER stress and contributes to paraptosis. Additionally, studies have indicated that the inactivation of the PI3K/AKT/mTOR signaling pathway is implicated in the process of paraptosis [2,3,4]. Therefore, the expression levels of genes involved in this signaling pathway were assessed in HCC cell lines treated with Ezetimibe. The results demonstrated that Ezetimibe dose-dependently inhibited the expression levels of p-PI3K, p-AKT, p-S6K1, and p-mTOR proteins (Figure 6B and Figure S5), suggesting that the downregulation of the PI3K/AKT/mTOR signaling pathway is also involved in Ezetimibe-induced paraptosis.

The mechanism by which Ezetimibe regulates the MAPK and PI3K/AKT/mTOR signaling pathways was investigated. It is known that Ezetimibe directly targets and binds to the NPC1L1 protein, inhibiting its function [41]. NPC1L1 is localized on the membranes of enterocytes and hepatocytes, where it mediates intestinal cholesterol absorption and carries biliary cholesterol into hepatocytes to prevent cholesterol loss [42]. To confirm that NPC1L1 is the target of Ezetimibe in HCC cells, NPC1L1 expression was measured in HCC cells treated with or without Ezetimibe using RT-PCR analysis. Comparing HCC cell lines treated with Ezetimibe to control HCC cells, NPC1L1 expression was significantly downregulated (Figure 6C), confirming that NPC1L1 is indeed a target of Ezetimibe in HCC cells. To further validate this, NPC1L1 was overexpressed in SNU-449 and HepG2 cells treated with Ezetimibe. The results showed that the cell viability inhibited by Ezetimibe was partially restored in HCC cells overexpressing NPC1L1 compared to those without NPC1L1 overexpression (Figure 6D and Figure S6A), demonstrating that Ezetimibe decreases cell survival by targeting NPC1L1. Moreover, compared with cells treated with Ezetimibe alone, the overexpression of NPC1L1 significantly reduced the cytoplasmic vacuolation induced by Ezetimibe in SNU-449 cells (Figure S6B), further supporting the notion that Ezetimibe induces cytoplasmic vacuolation by targeting NPCL1L1 in HCC cells.

The underlying mechanism by which Ezetimibe triggers ER stress and vacuolation was further elucidated by evaluating the PI3K/AKT/mTOR signaling pathway in HCC cells overexpressing NPC1L1 and treated with or without Ezetimibe. The results, as depicted in Figure 6E and Figure S7, demonstrated that the overexpression of NPC1L1 inhibited the expressions of BIP and p-PERK, which were related to ER stress, while enhancing the expression of PI3K, p-AKT, and p-mTOR in SNU-449, Huh-7, and HepG2 cells. These findings indicate that Ezetimibe induces ER stress by targeting NPC1L1 and inhibiting the PI3K/AKT/mTOR signaling pathway. To further confirm the role of the mTOR signaling pathway in HCC cells, treatment with an mTOR inhibitor (rapamycin) [43] and an mTOR activator (MHY1845) was performed. As shown in Figure 6F, co-treatment with Ezetimibe and rapamycin significantly decreased the expression levels of p-mTOR and noticeably increased the expression of BIP and CHOP protein compared to Ezetimibe treatment alone in SNU-449 and Huh-7 cells. Conversely, co-treatment with Ezetimibe and MHY1845 significantly attenuated the Ezetimibe-induced upregulation of BIP and elevated p-mTOR protein compared to Ezetimibe treatment alone in both SNU-449 and Huh-7 cells. Moreover, co-treatment with Ezetimibe and rapamycin further increased vacuolization, while co-treatment with Ezetimibe and MHY1845 significantly reduced the Ezetimibe-induced vacuolization in SNU-449 cells (Figure 6G). It is worth noting that neither rapamycin nor MHY1845 alone induced vacuolization. In summary, these findings demonstrate that Ezetimibe activates the MAPK signaling pathway and inhibits the mTOR signaling pathway by targeting NPC1L1 in HCC cells.

### 3.7. Ezetimibe Inhibits Growth of Hepatocellular Carcinoma Tumor in Xenograft Mouse Model

**Figure 7:**
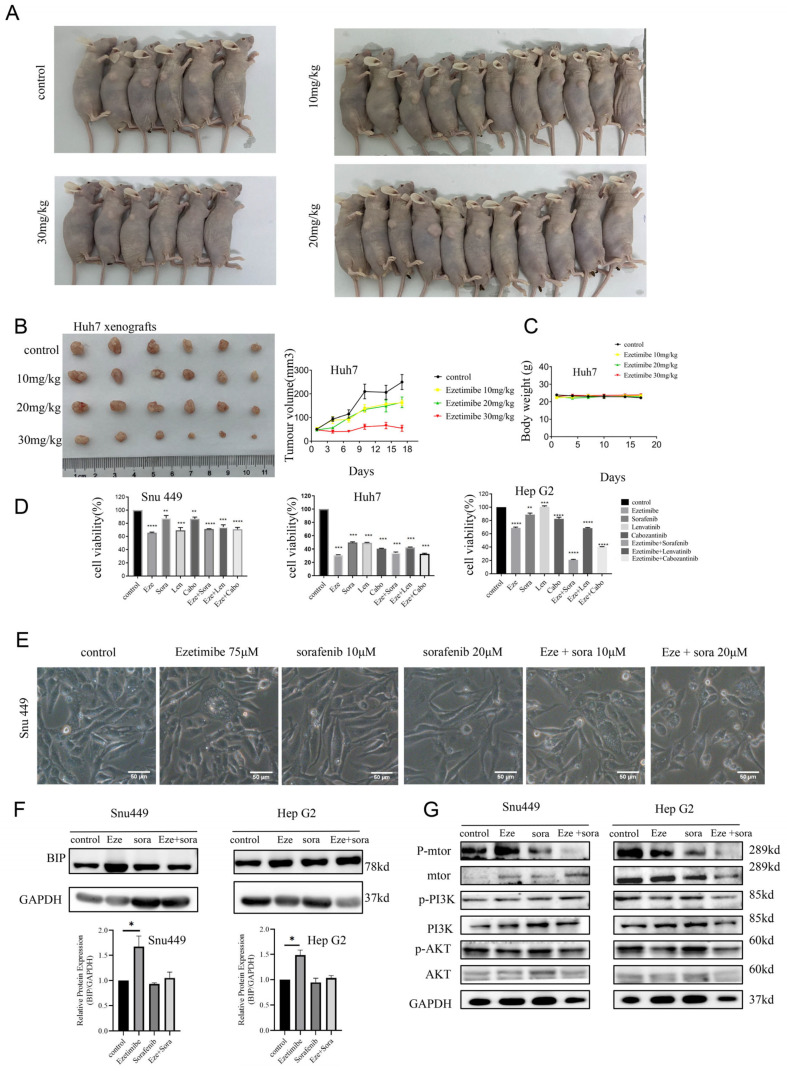
Ezetimibe inhibits tumor growth in mouse model. (**A**) After the experiment was completed, mice were euthanized. (**B**) The tumor volumes were measured once every 3 days, and growth curves of tumors are shown in photos of dissected tumors from the 4 mouse groups. (**C**) Curves of mouse body weight. (**D**) Cell viability was measured by CCK8 assay on HCC cells treated with Eze (Ezetimibe, 75 μM), Sora (Sorafenib, 10 μM), Len (Lenvatinib, 10 μM), Cabo (Cabozantinib, 10 μM), or combination of two agents. ** *p* < 0.01; *** *p* < 0.001; **** *p* < 0.0001. (**E**) Sorafenib had no impact on cytoplasmic vacuolization in SNU-449 cells. Scale bars, 50 μm. (**F**,**G**) The levels of proteins related to ER stress and PI3K/AKT/mTOR signaling were detected in HCC cells treated with Ezetimibe or/and Sorafenib, * *p* < 0.05.

We established an HepG2 xenograft mouse model of HCC to examine the tumor suppressor role of Ezetimibe. The mice were orally administered saline or Ezetimibe at doses of 10–30 mg/kg [44]. We measured the volume of tumor and body weight twice a week for a duration of 17 days, and upon euthanizing the mice, the tumors were harvested (Figure 7A,B). The results revealed that the tumors in mice treated with Ezetimibe were significantly smaller than those in the vehicle-treated mice, and the inhibitory effect of Ezetimibe on tumor growth was observed in a dose-dependent manner (Figure 7B). Notably, when administering 30 mg/kg of Ezetimibe, the tumor growth was nearly completely inhibited, as there was no increase in tumor size during the drug administration period. Throughout the experiment, no significant changes in body weight were observed among the different treatment groups (Figure 7C), indicating that Ezetimibe administration (up to 30 mg/kg) had no toxicity or side-effects in the mice.

Currently, the effectiveness of mono-targeted antitumor drugs in cancer patients is limited. However, reports have demonstrated that combining different targeted antitumor drugs can lead to improved therapeutic outcomes. Therefore, we further evaluated the effects of combining Ezetimibe with other FDA-approved anti-HCC drugs on HCC cells. Initially, HCC cells were treated with single agents or combinations of Ezetimibe with Sorafenib, Lenvatinib, or Cabozantinib. The results revealed that among all the single anti-HCC drugs, Ezetimibe exhibited the most potent inhibitory effect of cell viability on SNU-449, Huh-7, and HepG2 cells. Moreover, the combination of Ezetimibe with Sorafenib demonstrated the strongest inhibitory effect on HCC cells among the three combinations (Figure 7D). Furthermore, it was observed that only Ezetimibe induced cytoplasmic vacuolization in SNU-449 cells, while Sorafenib did not have this effect (Figure 7E), indicating different anticancer mechanisms of the two drugs. Additionally, Western blotting confirmed that treatment with Sorafenib did not enhance the expression of the ER-stress-related protein BIP in SNU-449 and HepG2 cells (Figure 7F). These results suggest that the co-treatment of Ezetimibe with Sorafenib significantly increased cell death in HCC cells through different anticancer mechanisms. By inhibiting the Raf/MEK/ERK pathway, Sorafenib reduces angiogenesis and induces apoptosis in tumor cells [45,46], and Sorafenib-acquired resistance is associated with abnormal activation of the PI3K/AKT/mTOR pathway [47]. We analyzed the activation of the AKT/mTOR signaling pathway using Western blotting. Indeed, the expression of p-mTOR and p-AKT was significantly downregulated in SNU-449 and HepG2 cells co-treated with Ezetimibe and Sorafenib compared to HCC cells treated with Ezetimibe or Sorafenib alone (Figure 7G and Figure S8). These findings collectively demonstrate that Ezetimibe has an anticancer role both in vivo and in vitro, and combining it with Sorafenib could be an effective treatment option for HCC patients.

## 4. Discussion

Inducing paraptosis only in the cancer cells as a therapeutic approach holds promise for selectively eliminating cancer cells as it targets mitochondrial and ER stresses. Currently, extensive research is focused on exploring the paraptosis-inducing potential of natural products in cancer cells. In our study, we investigated the anti-cancer effects of Ezetimibe, an effective lipid-lowering drug, both in vitro and in vivo. Promising anti-tumor effects of Ezetimibe were observed in an HCC xenograft mouse model derived from HepG2 cells at dosages ranging from 10 to 30 mg/kg. Our findings demonstrated that Ezetimibe induces excessive ROS generation and ER stress, ultimately leading to paraptosis in HCC cancer cells. Mechanistically, Ezetimibe activates the MAPK signaling pathway and inhibits the mTOR signaling pathway by targeting NPC1L1 in HCC cells.

A positive reciprocal regulation between ROS generation, mitochondrial Ca^2+^ overload, and ER stress has also been suggested. The disturbance of intracellular Ca^2+^ can trigger ER stress and mitochondrial dysfunction, impacting cellular proteostasis and contributing to paraptosis [48]. Additionally, mitochondrial Ca^2+^ overload induced by afatinib plus celastrol has been reported to be involved in paraptosis, leading to decreased ATP synthesis and AMPK phosphorylation [14,15,29]. Although mitochondria are not essential for inducing cytoplasmic vacuolization in paraptosis, m-tetra (hydroxyphenyl) chlorin has been found to induce ER photo-damage through paraptosis. In our current study, we discovered that Ezetimibe induces paraptosis by activating the ROS-mediated ER stress pathway, and the cell death induced by Ezetimibe can be reversed by the protein synthesis inhibitor CHX.

Resistance acquisition by HCC cells to Sorafenib is a major reason for the failure of cancer therapy with this drug. For instance, B3Z235 has been shown to increase the sensitivity of HCC cells to Sorafenib by inhibiting the PI3K/AKT/mTOR pathway and inducing autophagy [49]. Additionally, YB-1 (Y-box binding protein 1) enhances Sorafenib resistance in HCC by activating the PI3K/AKT signaling pathway, highlighting its association with drug resistance [3]. Copanlisib has been found to counteract Sorafenib-induced phosphorylation of AKT and synergistically enhance its antineoplastic effect [50]. Similarly, in our study evaluating the efficacy of Ezetimibe as a single or combination treatment, the combination of Ezetimibe and Sorafenib demonstrated the most effective results compared to other combinations. Interestingly, our results indicate that this combination enhances cell death by inhibiting the PI3K/AKT/mTOR pathway. However, further evidence based on HCC cells with acquired Sorafenib resistance is needed. Inherent or acquired drug resistance often leads to therapeutic failure and significant side-effects for patients, underscoring the urgent need for discovering new anti-cancer agents and developing innovative therapeutic approaches.

## Data Availability

The data and materials used in this research are available from the corresponding author upon reasonable request.

## Supplementary Materials

The following supporting information can be downloaded at https://www.mdpi.com/article/10.3390/genes15010004/s1. Figure S1: Ezetimibe inhibits proliferation of HCC cells. (A) Cell proliferation is gradually decreased in HCC cells treated with increasing concentrations of Ezetimibe for 48 h; cell viability was measured using CCK8 assay. (B) EDU staining was used to measure the cellular proliferation based on the reaction with Alexa488-azide (green). Scale bars, 50 μm. Figure S2: Ezetimibe induces ROS in HCC cells. Huh-7 cells were treated with Ezetimibe ranging from 0 to 75 μM for 24 h and then the cells were stained with DCFH-DA of Cellular ROS Assay Kit (ROS exhibited green fluorescence). Scale bars, 50 μm. Figure S3: Cycloheximide reduces Ezetimibe-induced vacuolation in HCC cells. Cycloheximide (CHX, 8 μM) significantly decreased perinuclear vacuolation induced by Ezetimibe at 24 h compared with cells treated with Ezetimibe alone. Scale bars, 20 μm. Figure S4. Ezetimibe upregulates the expression levels of MAPK pathway. Quantification of MAPK-signaling-pathway-related proteins and bar presentation. * *p* < 0.05, ** *p* < 0.01. Figure S5. Ezetimibe decreases the expression levels of mTOR pathway. Quantification of mTOR-signaling-pathway-related proteins and bar presentation. * *p* < 0.05, ** *p* < 0.01, *** *p* < 0.001. Figure S6: Ezetimibe induces paraptosis by targeting NPC1L1. (A) After transfecting with the over-expression plasmid, Western blot was used to determine the level of NPC1L1. (B) Overexpression of NPC1L1 could significantly eliminate vacuoles induced by Ezetimibe in SNU-449 cells. Scale bars, 50 μm. Figure S7. Overexpression of NPC1L1 inhibits the expressions of ER-stress-related proteins and enhances the expression of PI3K, p-AKT, p-mTOR in SNU449, Huh-7, and HepG2 cells lines. (A) Quantification of NPC1L1 protein and bar presentation. * *p* < 0.05, ** *p* < 0.01. (B) Quantification of expression of PI3K/AKT/mTOR and PERK proteins. * *p* < 0.05, ** *p* < 0.01, *** *p* < 0.001. Figure S8. Co-treatment of Ezetimibe and Sorafenib downregulates the expression of mTOR signaling pathway. Quantification of mTOR-signaling-pathway-related proteins and bar presentation. * *p* < 0.05, ** *p* < 0.01; Video S1: Control1; Video S2: Eztimibe1.
