# Supplementary material for: Ezetimibe Induces Paraptosis through Niemann–Pick C1-like 1 Inhibition of Mammalian-Target-of-Rapamycin Signaling in Hepatocellular Carcinoma Cells
Source: Genes (Basel). 2023 Dec 19;15(1):4. doi: 10.3390/genes15010004 (PMC10815321; doi:10.3390/genes15010004)
Supplement: Supplementary file 1 [file genes-15-00004-s001.zip › supplementary data.pdf]

Supplementary Data

Ezetimibe induces paraptosis via targeting NPC1L1 to inhibit mTOR/MAPK signaling pathway in hepatoma

Yu-ting Yin, et al

Supplementary Figures

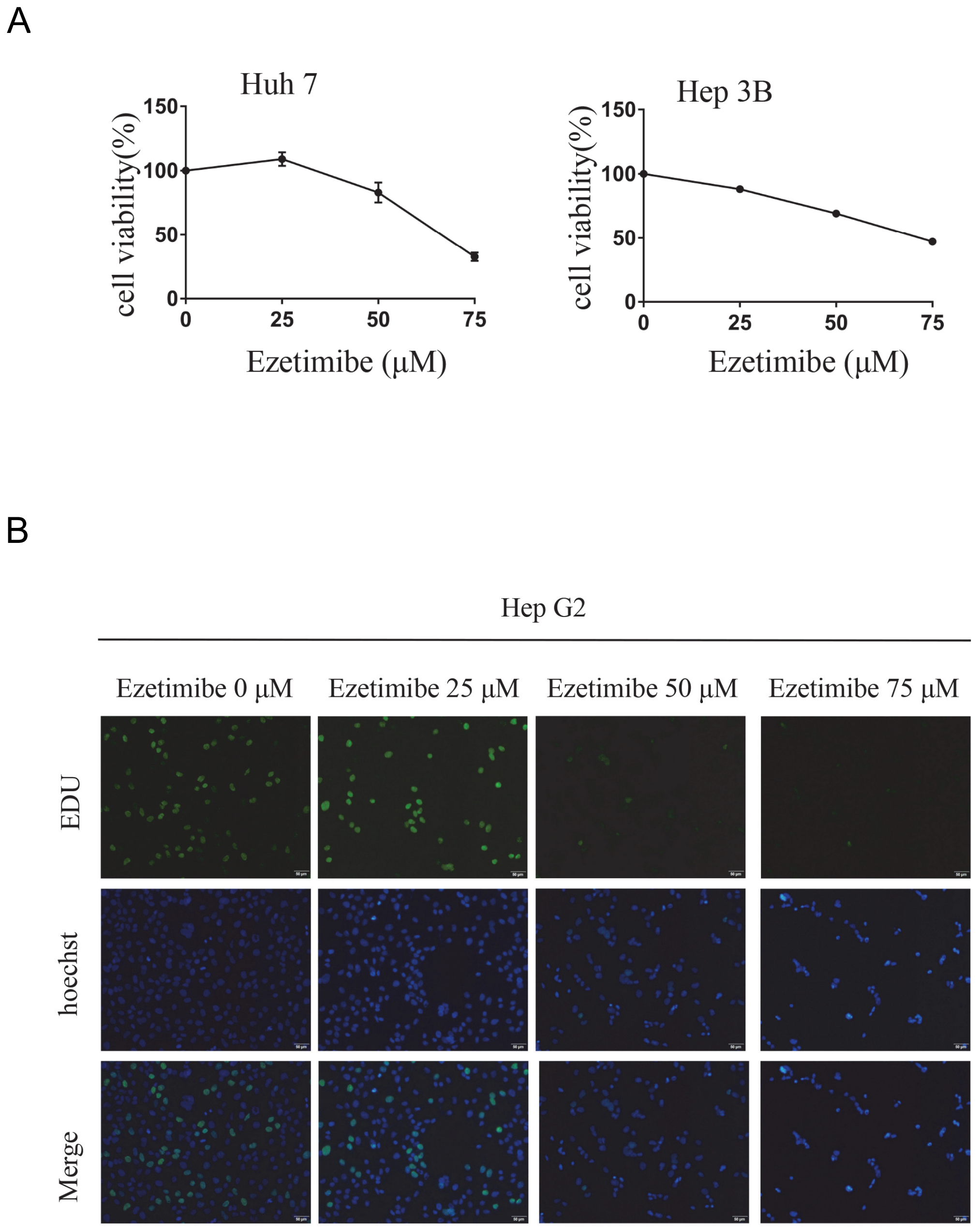

C

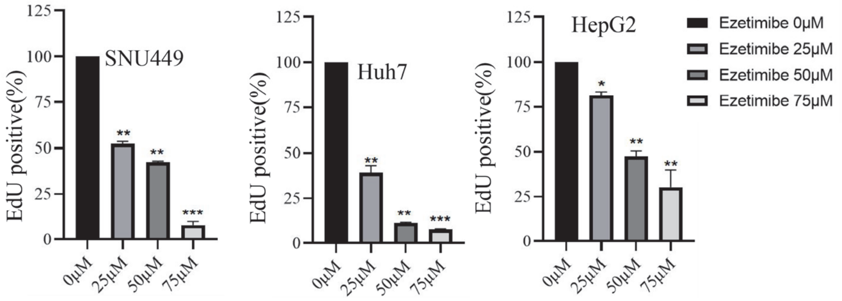

Fig. S1. **Ezetimibe inhibits proliferation of HCC cells.** (A) Cell proliferation is gradually decreased in HCC cells treated with increasing concentrations of Ezetimibe for 48h; cell viability was measured by CCK8 assay. (B) EDU staining was used to measure the cellular proliferation based on the reaction with Alexa488-azide (green). Scale bars, 50 μm. (C) Quantification of EdU-positive cells. $**P < 0.01$, $***P < 0.001$.

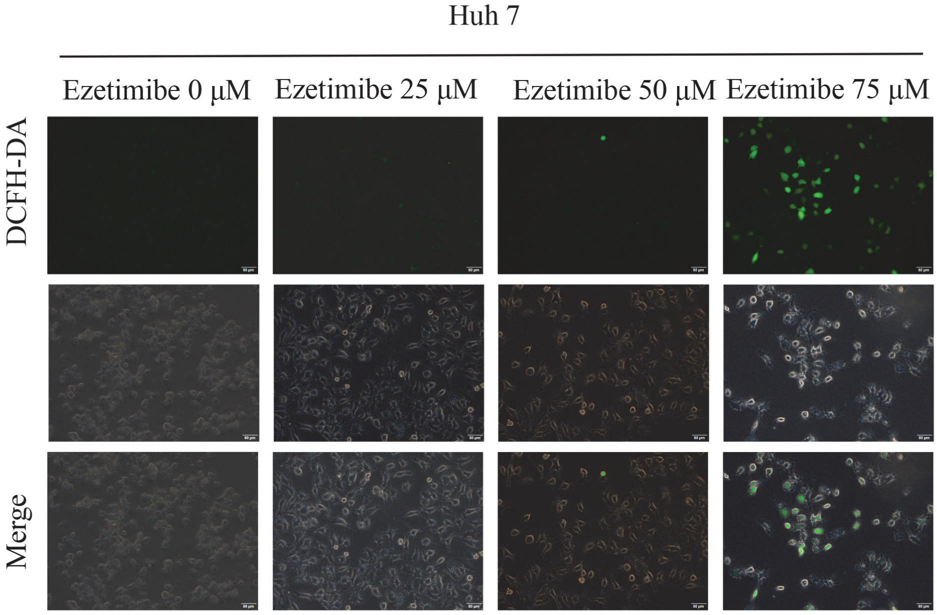

Fig. S2. Ezetimibe induces ROS in HCC cells. Huh-7 cells were treated with Ezetimibe ranging from 0 to 75μM for 24h and then the cells were stained with DCFH-DA of Cellular ROS Assay Kit (ROS exhibited green fluorescence). Scale bars, 50 μm.

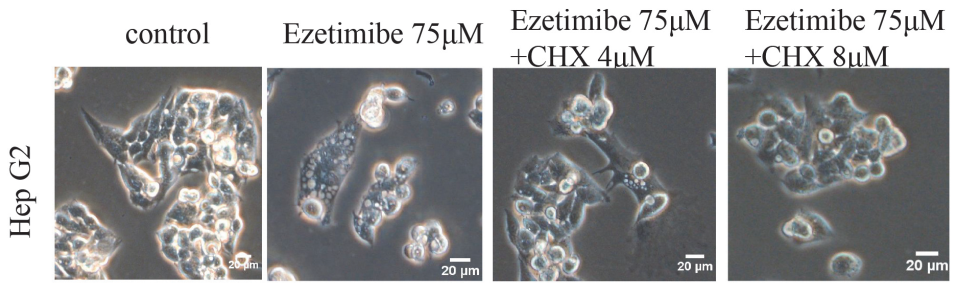

Fig. S3. Cycloheximide reduces Ezetimibe-induced vacuolation in HCC cells. Cycloheximide (CHX, 8μM) significantly decreased perinuclear vacuolation induced by Ezetimibe at 24h compared with cells treated with Ezetimibe alone. Scale bars, 20 μm.

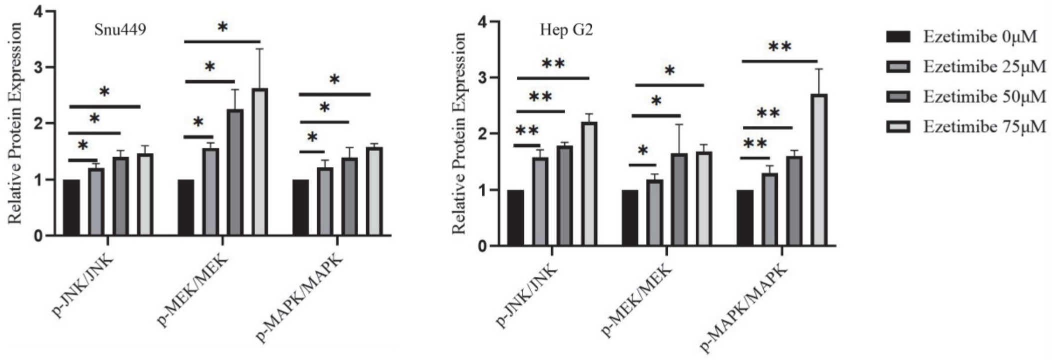

Fig.S4. Ezetimibe upregulates the expression levels of MAPK pathway. Relative level of MAPK signaling pathway expression was quantified by ImageJ software and bar presentation. *P < 0.05, **P < 0.01.

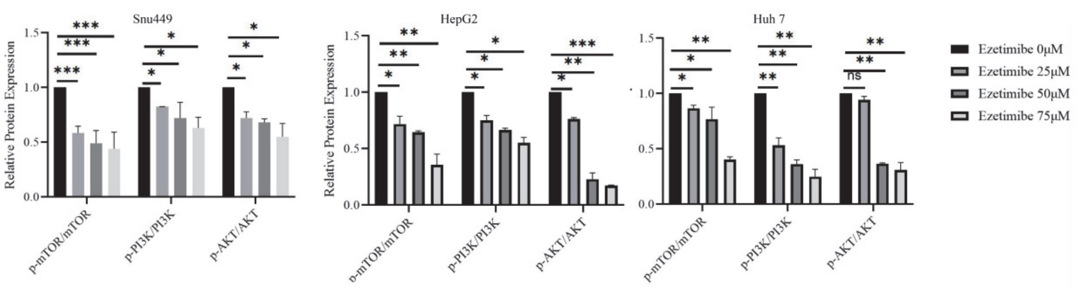

Fig.S5. Ezetimibe decreases the expression levels of mTOR pathway. Relative level of mTOR signaling pathway expression was quantified by ImageJ software and bar presentation. *P < 0.05, **P < 0.01, ***P < 0.001.

A

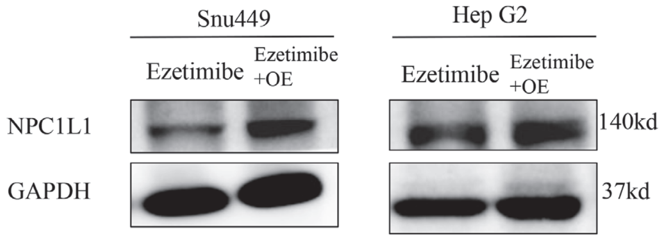

B

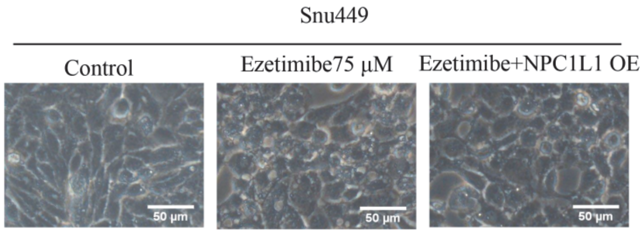

Fig. S6. Ezetimibe induces paraptosis by targeting NPC1L1. (A) After transfecting with the over-expression plasmid, Western blot was used to determine the level of NPC1L1. (B) Overexpression of NPC1L1 could significantly eliminate vacuoles induced by Ezetimibe in SNU-449 cells. Scale bars, 50 μm.

A

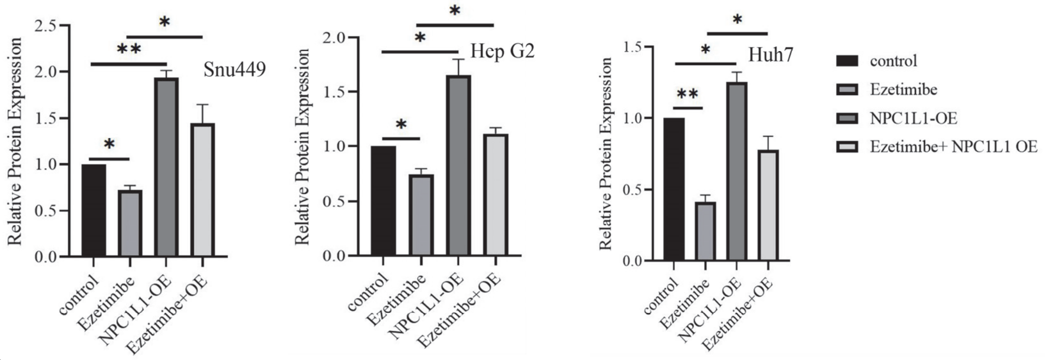

B

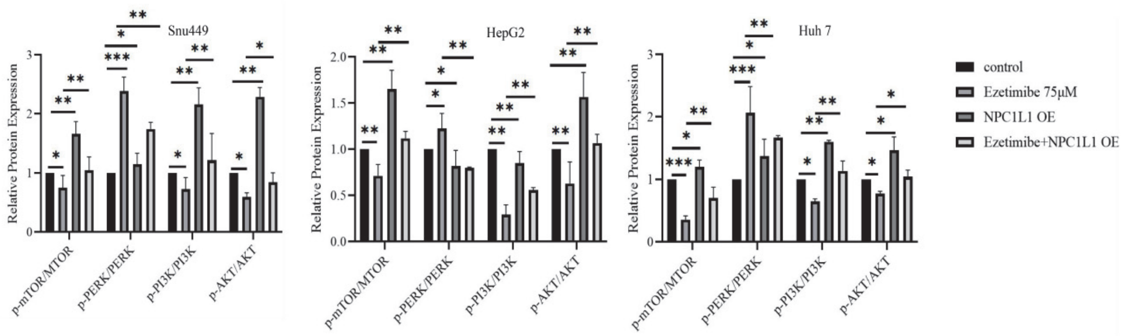

Fig. S7. Overexpression of NPC1L1 inhibits the expressions of ER stress-related proteins and enhances the expression of PI3K, p-AKT, p-mTOR in SNU449, Huh-7 and HepG2 cells lines. (A) Relative level of NPC1L1 expression was quantified by ImageJ software and bar presentation. *P < 0.05, **P < 0.01. (B) Quantification the expression of PI3K/AKT/mTOR and PERK proteins. *P < 0.05, **P < 0.01, ***P < 0.001.

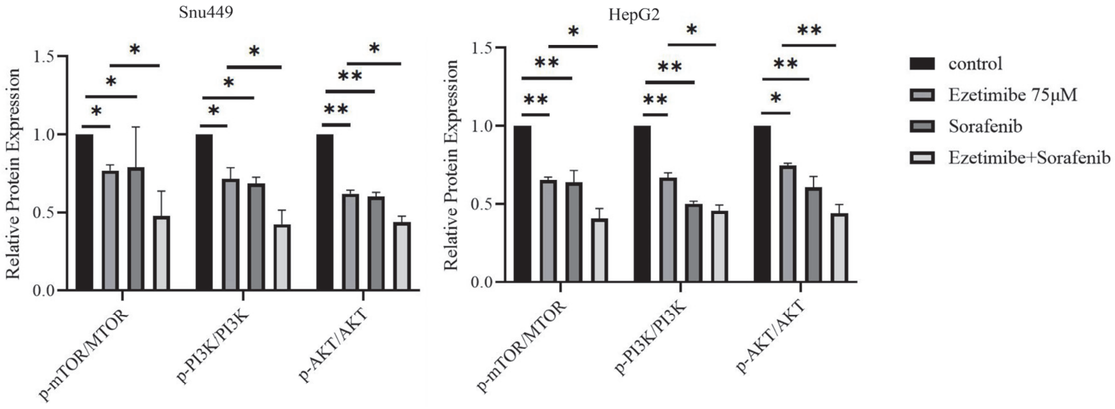

Fig.S8. Co-treatment of Ezetimibe and Sorafenibthe downregulates the expression of mTOR signaling pathway. Relative level of mTOR signaling pathway expression was quantified by ImageJ software and bar presentation. *P < 0.05, **P < 0.01.
